# My scientific genealogy and the Toronto ACDC Laboratory, 1988–2022

**DOI:** 10.1515/biol-2022-0483

**Published:** 2022-11-09

**Authors:** Eleftherios P. Diamandis

**Affiliations:** Department of Pathology and Laboratory Medicine, Mount Sinai Hospital, 60 Murray St [Box 32]; Flr 6 – Rm L6-201-1, Toronto, ON, M5T 3L9, Canada; Department of Laboratory Medicine and Pathobiology, University of Toronto, Toronto, Canada; Department of Clinical Biochemistry, University Health Network, Toronto, Canada

**Keywords:** scientific genealogy, mentorship, ACDC laboratory, most important scientific contributions, training

## Abstract

There is a saying that as people get older, they prefer to speak more about the past and less about the future. As I go through the last chapter of my scientific career, which spans from 1988–2022, I traced my scientific genealogy and the most important scientific achievements of my laboratory. By examining close to 1,000 PubMed-indexed papers published, I found out that none of them describes best our most important contributions. Also, by realizing that our contributions in science would have likely been discovered by others shortly afterwards, I focused my attention to other metrics. I suggest here that the best metric of success is the number of people that have been trained in my lab, and found their own way in their professional and other endeavors. Over the years, I trained over 250 individuals, of which 49 obtained a PhD, 19 an MSc, 37 were post-doctoral fellows, 5 were clinical fellows and about 150 were co-op/undergraduates and summer students. Many of these individuals now hold important positions in Academia, Government and Industry. My graduates, who have now created their own genealogy and many more individuals with roots to my laboratory, are now serving the society. In conclusion, I consider the development of young trainees as my most important career contribution.

## 1 Introduction

During this last chapter of my scientific endeavors, which span about 34 years, I frequently go back in time and reminisce on what my team has done and what has been achieved. My research laboratory was established in 1988 at the Toronto Western Hospital (now part of the University Health Network) and was given the catchy name “ACDC Laboratory” for reasons described in another essay [1]. ACDC stands for Advanced Center for Detection of Cancer and highlights our work on cancer biomarkers.

One question that frequently comes up is which ACDC Lab paper (excluding reviews), among the nearly 1,000 that are listed in PubMed, wins the imaginary prize of being the best work of my team.

Initially, I thought that our best paper should be the one with the most citations. We have a paper dating back to 1995, examining the ability of red wine phenolics to block platelet aggregation [2]. This paper consistently attracts over 55 citations per year and as of today, has a total of >1,400 citations. It was published in a relatively low impact journal, *Clinica Chimica Acta*, with a current Impact Factor of 3.8. For comparison, I mention here that the average citations of a paper published in the prestigious journals *Nature or Science* is about 30–40 per year. Although this is our most cited paper, I doubt, by comparison to our other papers, that its content is also the most significant.

My second thought went to a paper that was published in 2013 in the prestigious journal *Science Translational Medicine*, describing discovery and validation of a new semen biomarker of male infertility, TEX101 [3]. Despite its publication in a prestigious journal and the licensing of the intellectual property for commercial/clinical use by American and Chinese companies, this paper is currently receiving only 15 citations per year.

My third choice was a paper that in my strong view had the most impactful scientific value and included for the first time the molecular characterization of the whole human kallikrein locus on chromosome 19q13.4 [4]. Kallikreins are 15 homologous serine proteases. Although the content of this paper is widely considered as a milestone in the field of kallikrein proteases, the paper was originally declined for publication by the legendary *Journal of Biological Chemistry* and it was published in a relatively low impact journal, Biochemical and Biophysical Research Communications (current Impact Factor 3.5) is attracting a modest number of citations (11 citations per year). Instead of this original paper, authors who publish in the kallikrein field now prefer to cite other papers and reviews of our group on this subject [5,6]. This “secondary referencing” is common practice in science.

### 1.1 The obsession of being first

After commenting on scientific discoveries, citations and journals with high or low impact, I also for long realized that even papers with very high scientific value, may not, in the long run, make much difference in the progression in science. Let us take as an example the seminal paper published in 1953 from Watson and Crick in Nature, which was a one-page report on the double helix structure of DNA [7]. Although this paper is highly acclaimed, and considered one of the landmarks of modern science, it is hard to believe that the double helix would have not been discovered 1 or 2 years later by other investigators. Along the same token, I tend to believe that our own “notable” contributions in science, involving the discovery of the human kallikrein locus and the numerous associated findings, such as biomarker and therapeutic applications [8,9], would have been unraveled by others, 1 or 2 years down the line. Another serendipitous and unexpected finding of our team in the late 1990s, the discovery of prostate specific antigen (PSA) in female breast [10], became obvious when scientists sequenced the transcriptome of breast cancer cell lines and found PSA transcripts in abundance.

Scientists have an obsession of being first to make a discovery. This rather arrogant behavior falls within the realm of competitive science, which has a scope of facilitating accelerated progress. However, I believe that even the most ingenious discoveries, eventually, are destined to be uncovered by others sometime later, and are likely to have similar impact on humankind in the long run. A good analogy could be the development of the smartphone. Would it make a lot of difference if humans, who lived without smartphones for centuries, could have waited 2–3 more years to use them? I believe not.

Because of the above, I have started diverting my views on what are my lab’s major contributions to science, by looking at indicators outside of our published work. I came to the conclusion that probably the most important contributions of the ACDC Lab are in education, mentoring and in helping to build the careers of others. Over the years, hundreds of people passed through the lab and trained at all levels from PhD to Masters, to Post-doctoral or technical work. To obtain an accurate picture, I decided to track as many of our ex-members as possible, and see where they are now, and what they achieved in their own careers. I included all classes of trainees and other staff, and summer students, since even the latter acquired some tools that helped them move forward their careers.

In the attached Table 1, I cite cumulative numbers of ex-staff and mention the highest rank achieved in their chosen profession, after leaving the lab. Obviously, we were not able to track all of our past staff, especially the group of summer students, whose career is still evolving. A few interpretative comments are useful. I selected ten arbitrary professional categories which may or may not accurately represent the profession of everybody listed. Also, many individuals fall into more than one category, such as Professor and Physician-MD. In such case, I selected only one category, to avoid over-estimations.

**Table 1:** Categorization of my scientific collaborators/trainees (*n* = 253) who worked at the ACDC laboratory from 1988–2022

Current/highest professional position	Graduate students PhD	Graduate students MSc	Postdocs	Medical residents	Undergraduate-co-op students	Summer students
Professor	12	1	5		0	1
Physician-MD	6	10	1	5	2	26
Government administrator	0	0	0			2
Research associate	5	1	0			4
Industry scientist	9	3	7			3
Entrepreneur	1	0	0			2
Academic scientist	4	1	10			0
Academic administrator	3	1	0		1	3
Clinical chemist	6	0	6	0		0
Technologist/manager	0	0	0		1	1
Unknown/evolving profession	3	2	8		10	90
Total numbers	49	19	37	5	14	129

These individuals are stratified by degree/position and by their highest profession achieved after leaving the lab. Professional groups are arbitrary and may not necessarily reflect accurately the profession of our trainees.

### 1.2 Findings

The ACDC Lab completed 49 PhD theses. The largest category of graduates (*n* = 12) are professors, followed by industrial scientists (*n* = 9) and physicians (*n* = 6). Among the 19 MSc graduates, 10 became physicians, confirming the view that many MSc candidates use this degree as a stepping stone to MD degree. Among the 37 postdocs, the largest group (*n* = 10) became academic scientists, followed by industrial scientists (*n* = 7) and professors (*n* = 5). Although we could not track most undergraduate and summer students, it is clear that at least the latter group is seeking entry into medical schools and this is consistent with my personal discussions with them. The success rate is around 25%.

Among the whole group (*n* = 253), 12 are practicing Clinical Biochemists and 6 of those, completed a PhD in the ACDC Lab before they entered the post-doctoral training program.

### 1.3 Person-years

If I assign average times for PhD (5 years), MSc (2 years) postdoctoral training (3 years), medical residency (1 year), undergraduate training (1 year) and summer studentship (0.25 years), the cumulative person-years in the ACDC Lab is 245 + 38 + 111 + 5 + 14 + 32 = 445 years.

We still have relationships with many of my ex-trainees. Many of our diverse activities, scientific or non-scientific, including music, sports, etc., can be found on our website (www.acdclab.org).

The lab gave the opportunity to some, to create productive personal relationships and we had a few weddings between lab members and several children were born as a result.

### 1.4 Who cares?

You may wonder as to why I, or others, would care for this information. The answer is simple. I care, for the same reason Elon Musk cares about how many billions of dollars he has in the bank. These dollars are his lifetime investments and the information provided are my own lifetime investments. Why would others care? For the same reason that these others, care as to who are the richest people on earth. While the Musks and the Bezos of the world care about money, I care about my human investments. Investing in humans should be a much more interesting exercise than investing in cars or Amazon deliveries!
